# Risk scores for predicting incident chronic kidney disease among rural Chinese people: a village-based cohort study

**DOI:** 10.1186/s12882-020-01787-9

**Published:** 2020-04-06

**Authors:** Jiangping Wen, Jie Hao, Ye Zhang, Kai Cao, Xiaohong Zhang, Jiang Li, Xinxin Lu, Ningli Wang

**Affiliations:** 1 Department of Laboratory Medicine, Beijing Tongren Hospital, Capital Medical University, No. 1 Dongjiaominxiang, Beijing, 100730 Dongcheng District China; 2 Beijing Tongren Eye Center, Beijing Tongren Hospital, Capital Medical University, Beijing Ophthalmology and Visual Science Key Laboratory, No. 1 Dongjiaominxiang, Beijing, 100730 Dongcheng District China; 3 Beijing Institute of Ophthalmology, No. 1 Dongjiaominxiang, Beijing, 100730 Dongcheng District China; 4 Department of Laboratory Medicine, China-Japan Friendship Hospital, No. 2 Yinghuayuan East Street, Beijing, 100029 Chaoyang District China

**Keywords:** Risk score, Chronic kidney disease, Rural China

## Abstract

**Background:**

Few chronic kidney disease (CKD) risk prediction models have been investigated in low- and middle-income areas worldwide. We developed new risk scores for predicting incident CKD in low- and middle-income rural Chinese populations.

**Methods:**

Data from the Handan Eye Study, which was a village-based cohort study and conducted from 2006 to 2013, were utilized as part of this analysis. The present study utilized data generated from 3266 participants who were ≥ 30 years of age. Two risk models for predicting incident CKD were derived using two-thirds of the sample cohort (selected randomly) using stepwise logistic regression, and were subsequently validated using data from the final third of the sample cohort. In addition, two simple point systems for incident CKD were generated according to the procedures described in the Framingham Study. CKD was defined as reduced renal function (estimated glomerular filtration rate (eGFR) < 60 mL/min/1.73m^2^) or the presence of albuminuria (urinary albumin-to-creatinine ratio (UACR) ≥30 mg/g).

**Results:**

The Simple Risk Score included waist circumference, systolic blood pressure (SBP), diabetes, sex, and education. The Best-fit Risk Score included urinary albumin-to-creatinine ratio, SBP, C-reactive protein, triglyceride, sex, education, and diabetes. In the validation sample, the areas under the receiver operating curve of the Simple Risk Score and Best-fit Risk Score were 0.717 (95% *CI*, 0.689–0.744) and 0.721 (95% *CI*, 0.693–0.748), respectively; the discrimination difference between the score systems was not significant (*P* = 0.455). The Simple Risk Score had a higher Youden index, sensitivity, and negative predictive value, with an optimal cutoff value of 14.

**Conclusions:**

Our Simple Risk Score for predicting incident CKD in a low- and middle-income rural Chinese population will help identify individuals at risk for developing incident CKD.

## Background

Chronic kidney disease (CKD) is strongly associated with an increased risk of developing end-stage renal disease, cardiovascular disease (CVD), and death [1]. Epidemiological studies have shown that the prevalence of CKD varies across countries and regions, including developed and developing areas [1–6]. CKD is highly prevalent in low- and middle-income areas [2, 3, 7, 8]. In China, a recent national survey reported that the prevalence of CKD was 10.8% and the number of patients with CKD was estimated to be about 119.5 million; however, awareness of CKD was only 12.5% [2]. Therefore, CKD prevention has become a major public health issue in China.

Predicting individual risk is the first step in the primary prevention of CKD. Risk scores that can identify those at higher risk for future CKD have been proposed as prediction and stratification methods [9, 10]. Several risk scores for predicting incident CKD have been developed and validated in Western populations [11–14]. These risk scores are based on clinical and laboratory information and have been suggested for use as tools to screen individuals considered to be high-risk for developing CKD in developed countries. However, a recent study showed that there is a higher prevalence of early-stage CKD and a lower prevalence of decreased renal function in China compared to the US [4]. Possible explanations for these variations include differences in ethnicities, socioeconomic statuses, risk factors, and genetic susceptibilities to renal disease [4]. Therefore, an ethno- or region-specific risk score for incident CKD was needed. Further, China, as the world’s largest developing country, has experienced a rapid increase in the prevalence of diabetes, hypertension, and obesity [2, 15]. To date, several cross-sectional studies have reported the prevalence of CKD and associated risk factors in Chinese populations [2, 8, 16]; however, a tool for predicting the risk of developing CKD in Chinese populations living in low- and middle-income areas had not been developed.

In China, screening for CKD should be a priority in low- and middle-income areas, because early intervention is likely to be effective in reducing the high morbidity and mortality rates resulting from CKD. In this study, we aimed to develop a simple risk score for predicting incident CKD in a population living in a low- and middle-income rural area of China. This CKD scoring system is simple and can be integrated into the rural primary health care system and help screen individuals that may be at risk for CKD.

## Methods

### Study population

We used the data from the Handan Eye Study (HES). Details of the rationale, design, methods and procedures related to this study were provided in our previous reports [17, 18]. The HES was a village-based cohort study to investigate eye diseases and other health-related problems among general rural residents aged ≥30 years old living in Yongnian County (a rural county of Handan City and located about 500 km south of Beijing). In this area, 80% of the population is engaged in agricultural production, 98% are Han people, and the net income per capita is 3468 yuan (approximately 468 USD), which is equivalent to the average income of the residents of China (3587 Yuan, 484 USD) [18]. This study was carried out in accordance with the Helsinki Declaration and approved by the Ethics Committee of Beijing Tongren Hospital (approval number # TREC2006–22). All subjects provided written informed consent. The right forefinger stamp was considered a signature substitute for illiterate people, which has been approved by the Ethics Committee.

**Fig. 1:**
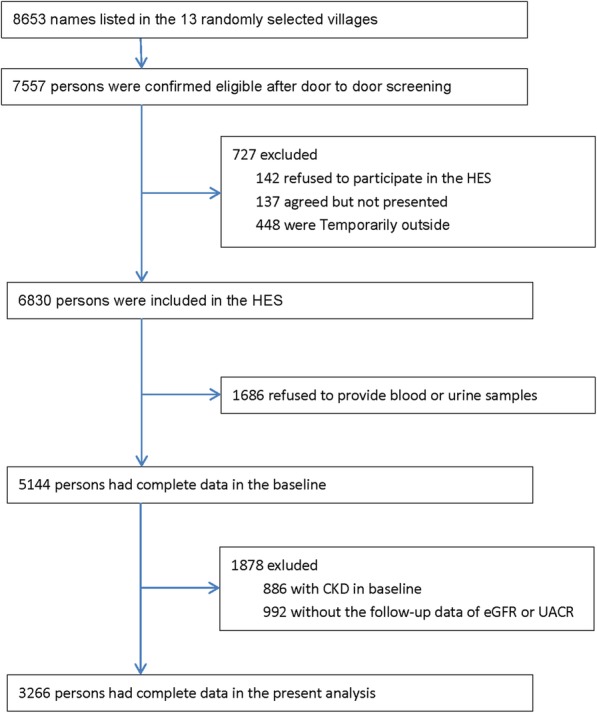
Flow diagram of participant recruitment

As shown in Fig. 1, 7557 of the 8653 subjects screened were considered eligible for HES. A total of 6830 participants participated in HES from October 2006 to October 2007, and a follow-up survey was carried out from May 2012 to June 2013 [17]. At baseline, 1686 participants declined to provide blood or urine samples, and 886 participants who were diagnosed with CKD were excluded. CKD was defined by reduced renal function (estimated glomerular filtration rate [eGFR] < 60 mL/min/1.73 m^2^) or albuminuria (urinary albumin-to-creatinine ratio [UACR] ≥30 mg/g) [19]. In follow-up, 992 individuals did not have available eGFR or UACR data. Consequently, 3266 participants were incorporated into the final analysis (Fig. 1).

### Data collection

The survey was carried out in local rural health centers. Interviewers with standard training obtained demographic information through questionnaires, including birth date, sex, ethnicity, occupation, education, smoking, drinking, physical activity, dietary habits and medical history. According to the number of years of education, they were divided into four groups (illiterate for 0 years, primary school for 1–6 years, junior high school for 7–9 years, and senior high school for ≥10 years). Physical activity was divided into three groups, including low exercise (little or no exercise), moderate exercise (walking or bicycling for more than 10 min, 1–3 times a week) and high exercise (leading to rapid breathing for more than 10 min, more than 3 times a week). Smoking and drinking were separated into three groups (never used, current user, and former user). Dietary habits included two categories (fresh fruit and vegetables). Consumption of fresh fruit and vegetables was divided into four frequency levels: ≥3 times per week, 1–2 times per week, 1–3 times per month, and never/very little.

During medical examinations, participants took two blood pressure measurements using a non-invasive automatic HEM-907 blood pressure monitor (OMRON, Kyoto, Japan) after 5 minutes of rest. Systolic blood pressure (SBP) and diastolic blood pressure (DBP) were identified as the average values of two independent measurements. Body mass index (BMI) was calculated by weight (kg) /height (m^2^).

All participants were asked to fast for at least 8 hours before drawing blood, which was taken in the morning in their respective villages. Serum creatinine was determined by the Jaffé kinetic method, fasting blood glucose (FPG) by the hexokinase method and serum lipid by the enzymatic method (Olympus AU27 00, Tokyo, Japan). Urinary albumin and creatinine were measured from fresh morning spot urine samples. Urinary albumin was determined by immunoturbidimetry (Holzheim Diasys Diagnostic Company, Germany). Urinary creatinine was determined by the same method as serum creatinine.

The HES baseline survey was carried out from October 2006 to October 2007, and the follow-up survey was carried out from May 2012 to June 2013. The baseline and follow-up surveys were carried out in local rural health centers, and CKD related data were collected at baseline and follow-up, seperately. CKD related data included demographic information, blood pressures, anthropometric measurements, serum and urine creatinine, and urinary albumin. CKD was defined by reduced renal function or the presence of albuminuria. Reduced renal function was defined by an eGFR < 60 mL/min/1.73 m^2^ and albuminuria was defined by a UACR ≥30 mg/g. The GFR estimation equation included serum creatinine, age and gender.

### Definitions of diabetes, hypertension, and CVD

Diabetes was defined as: (1) FPG ≥7.0 mmol/L, or (2) self-reported diagnosis of diabetes, or (3) the use of anti-diabetic medications [20]. Hypertension was defined as: (1) SBP ≥140 mmHg, or (2) DBP ≥90 mmHg, or (3) the use of antihypertensive medications [21]. CVD was defined as self-reported coronary heart disease, stroke, peripheral artery disease, or ankle-brachial index < 0.9 in either leg.

### Definition of CKD

CKD was defined by reduced renal function or the presence of albuminuria [19]. Albuminuria was defined by a UACR ≥30 mg/g. Because serum creatinine was measured via the Jaffé kinetic method, the modified Chinese equation was used [22]. Reduced renal function was defined by an eGFR < 60 mL/min/1.73 m^2^, calculated as follows:

eGFR = 175 × (Scr_Jaffe_)^−1.234^ × (Age in years)^−0.179^(×0.79 for women), where Scr indicates serum creatinine concentration (in mg/dL).

### Statistical analysis

In this study, SPSS v.18.0 software (IBM Corp., Chicago, IL, USA) was used for statistical analysis. The current analysis was limited to 3266 subjects with complete CKD data. The baseline characteristics of the subjects were described according to CKD status at follow-up. The means (standard deviations) or medians (interquartile ranges) were used for continuous variables, and the counts and percentages were used for categorical variables. Unpaired t-test or Mann-Whitney U-test were used to compare the average or median values, and chi-square test was used to analyze the categorical variables.

In this study, two-thirds of the samples were randomly selected as training samples, and the risk factors associated with incident CKD were investigated by forward stepwise logistic regression. CKD definition was a binary outcome with a cutoff of eGFR < 60 mL/min/1.73 m^2^ or UACR ≥30 mg/g. Based on previous studies, we identified a number of candidate risk factors, including age, sex, blood pressure, BMI, waist circumference (WC), smoking, alcohol consumption, education level, physical activity, diabetes, hypertension and CVD. In final models, only statistically significant risk factors were retained.

According to the methods described by Sullivan and colleagues in the Framingham risk score study [23], we developed a simple scoring system to estimate the risk of CKD. Firstly, continuous variables were classified and the reference values of each variable were defined separately. Secondly, the median value of each category was determined and the difference between each category and the reference in regression units was calculated. Thirdly, beta regression coefficients of continuous variables and classified variables are calculated, and the constant reflecting the increase of risk associated with WC or UACR was set. Finally, the score of each predictor was calculated by the product of the corresponding regression coefficients and the difference between the median of each predictor and the relevant reference group. The total scoring range was estimated according to the scoring calculated by each predictor.

After establishing the scoring system, we assessed its diagnostic ability for the remaining one-third of the samples (test samples). The sensitivity and 1-specificity of each cut point was used to plot the receiver operating characteristic curve. The areas under the receiver operating characteristic curve (AUC) were calculated based on the current risk scores. The predictive accuracy of the risk scoring systems can be assessed according to the AUC. We used Horsmer-Lemeshaw test to estimate the calibration characteristics of predictive scores. One of the non-significant *P* values indicates that there was a good consistency between the observed results and the model-based predictions. The optimal cutoff point of each risk scoring system was that the sum of sensitivity and specificity was the maximum. In addition, sensitivity, specificity, positive and negative predictive values, positive and negative likelihood ratio and Youden index were calculated. A two-sided *P* value < 0.05 was considered statistically significant.

## Results

### Baseline characteristics

As shown in Fig. 1, a total of 6830 participants participated in HES. At baseline, 1686 participants declined to provide blood or urine samples, and 886 participants who were diagnosed with CKD were excluded. The current analysis was limited to 3266 participants with complete CKD data. As shown in Table 1, at baseline, the proportion of women was 55.3%, illiterate or primary school education was 64.4%, regular physical activity was 69.3%, and hypertension was 45.6%. Compared to those without incident CKD, the participants who developed CKD were more likely to be women, to have a history of hypertension and diabetes, and to be taking antihypertensive agents; however, they tended to drink and smoke less, consumed fewer fresh fruits, and had lower education levels. They also were older and had higher BMIs, WCs, blood pressures, FPGs, total cholesterol levels, triglyceride levels, UACRs, and C-reactive protein (CRP) levels, but their eGFRs were lower.

**Table 1:** Baseline characteristics of participants with and without incidentchronic kidney disease (CKD) at follow-up

Characteristic	Total (*n* = 3266)	Without incident CKD (*n* = 2676)	With incident CKD (*n* = 590)	*P* value
Age (years)	50 ± 10	50 ± 10	53 ± 10	< 0.001
Female sex, n (%)	1805 (55.3)	1392 (52.0)	413 (70.0)	< 0.001
BMI (kg/m^2^)	24.6 ± 3.5	24.5 ± 3.5	25.1 ± 3.4	< 0.001
Waist circumference (cm)	87.2 ± 9.3	86.8 ± 9.2	89.1 ± 9.5	< 0.001
SBP (mmHg)	137.2 ± 20.7	134.9 ± 19.5	147.5 ± 22.7	< 0.001
DBP (mmHg)	77.3 ± 11.7	76.4 ± 11.2	81.1 ± 13.0	< 0.001
Total cholesterol (mmol/L)	4.56 ± 0.91	4.53 ± 0.90	4.72 ± 0.95	< 0.001
HDL-cholesterol (mmol/L)	1.27 ± 0.28	1.28 ± 0.28	1.26 ± 0.31	0.167
LDL-cholesterol (mmol/L)	2.68 ± 0.63	2.65 ± 0.63	2.80 ± 0.64	< 0.001
Triglycerides (mmol/L)	1.24 (0.87–1.79)	1.20 (0.85–1.73)	1.42 (0.98–2.08)	< 0.001
Fasting plasma glucose (mmol/L)	5.49 (5.16–5.89)	5.48 (5.14–5.86)	5.58 (5.24–6.08)	< 0.001
CRP (mg/L)	0.80 (0.37–2.05)	0.75 (0.35–1.88)	1.17 (0.49–2.75)	< 0.001
Urea (mmol/L)	4.78 ± 1.19	4.77 ± 1.17	4.82 ± 1.27	0.340
Creatinine (mmol/L)	70.9 ± 9.9	71.1 ± 9.8	70.2 ± 10.3	0.081
eGFR, ml/min/1.73 m^2^	102.6 ± 16.1	103.3 ± 16.3	99.6 ± 14.8	< 0.001
Albumin-to-creatinine ratio (mg/g)	6.89 (3.42–12.60)	6.60 (3.30–12.10)	8.52 (4.32–15.33)	< 0.001
Current smoker, n (%)	890 (27.3)	786 (29.4)	104 (17.6)	< 0.001
Current drinker, n (%)	610 (18.7)	543 (20.3)	67 (11.4)	< 0.001
Regular consumption of fresh fruits, n (%)	315 (9.6)	280 (10.5)	35 (5.9)	0.006
Regular consumption of fresh vegetables, n (%)	3245 (99.4)	2659 (99.4)	586 (99.3)	0.907
Education, n (%)				< 0.001
Illiterate	398 (12.2)	280 (10.5)	118 (20.0)	
Primary School	1704 (52.2)	1392 (52.0)	312 (52.9)	
Junior high	1062 (32.5)	915 (34.2)	147 (24.9)	
Senior high	102 (3.1)	89 (3.3)	13 (2.2)	
Physical activity, n (%)				0.856
Low	595 (18.2)	483 (18.0)	112 (19.0)	
Moderate	409 (12.5)	337 (12.6)	72 (12.2)	
High	2262 (69.3)	1856 (69.4)	406 (68.8)	
Hypertension, n (%)	1488 (45.6)	1099 (41.1)	389 (65.9)	< 0.001
Diabetes, n (%)	161 (4.9)	88 (3.3)	73 (12.4)	< 0.001
Cardiovascular disease, n (%)	234 (7.2)	185 (6.9)	49 (8.3)	0.235
Use of antihypertensive agents, n (%)	600 (18.4)	409 (15.3)	191 (32.4)	< 0.001
Use of lipid-lowering agents, n (%)	58 (1.8)	45 (1.7)	13 (2.2)	0.385
Use of anti-diabetic agents, n (%)	44 (1.3)	23 (0.9)	21 (3.6)	< 0.001

Data are presented as means ± standard deviations, medians (interquartile ranges), or numbers (percentages). Chi-square test was used for categorical variables and the unpaired t test or Mann-Whitney U test was used for continuous variables

*CKD* Chronic kidney disease, *BMI* Body mass index, *SBP* Systolic blood pressure, *DBP* Diastolic blood pressure, *HDL-C* High density lipoprotein cholesterol, LDL-C Low density lipoprotein cholesterol, *CRP* C-reactive protein, *eGFR* Estimated glomerular filtration rate

### CKD incident rates

Of the 3266 participants who presented without CKD at baseline, 590 (18.1%) developed CKD during a median of 5.6 years of follow-up. Of these, 565 (95.8%) participants with CKD were identified by the presence of albuminuria (UACR ≥30 mg/g) and 38 (6.4%) had reduced renal function (eGFR < 60 mL/min/1.73 m^2^).

### Risk models for predicting incident CKD

As shown in Table 2, two risk models for incident CKD were derived in the forward stepwise multivariable logistic regression analysis in the training population. The factors significant in the simple clinical model were sex, WC, SBP, diabetes, and education. The best-fit model included sex, SBP, diabetes, education, triglyceride, UACR, and CRP. The AUCs of the simple and best-fit models were 0.714 (95% confidence interval [CI], 0.686–0.742) and 0.725 (95% CI, 0.697–0.752) in the training samples, respectively.

**Table 2:** Stepwise logistic regression analyses of risk factors for incident CKD in the training population

	Variables	β-Coefficient	Odd ratios (95% CI)	***P*** value
**Simple clinical model**	Intercept	−6.168		< 0.001
	Waist circumference	0.013	1.01 (1.00–1.03)	0.045
	Sex	0.686	1.99 (1.56–2.53)	< 0.001
	Education	−0.532	0.59 (0.43–0.80)	0.001
	Diabetes	1.042	2.83 (1.85–4.33)	< 0.001
	SBP	0.025	1.03 (1.02–1.03)	< 0.001
**Best-fit model**	Intercept	−5.530		
	Sex	0.652	1.92 (1.50–2.45)	< 0.001
	Education	−0.516	0.60 (0.44–0.81)	0.001
	Diabetes	0.923	2.52 (1.64–3.86)	< 0.001
	SBP	0.025	1.03 (1.02–1.03)	< 0.001
	Triglycerides	0.175	1.19 (1.07–1.32)	0.001
	CRP	0.027	1.03 (1.01–1.05)	0.011
	Albumin-to-creatinine ratio	0.017	1.02 (1.00–1.03)	0.030

*CKD* Chronic kidney disease, *SBP* Systolic blood pressure, *CRP* C-reactive protein, *CI* Confidence interval

### Development of risk scores for predicting incident CKD

**Fig. 2:**
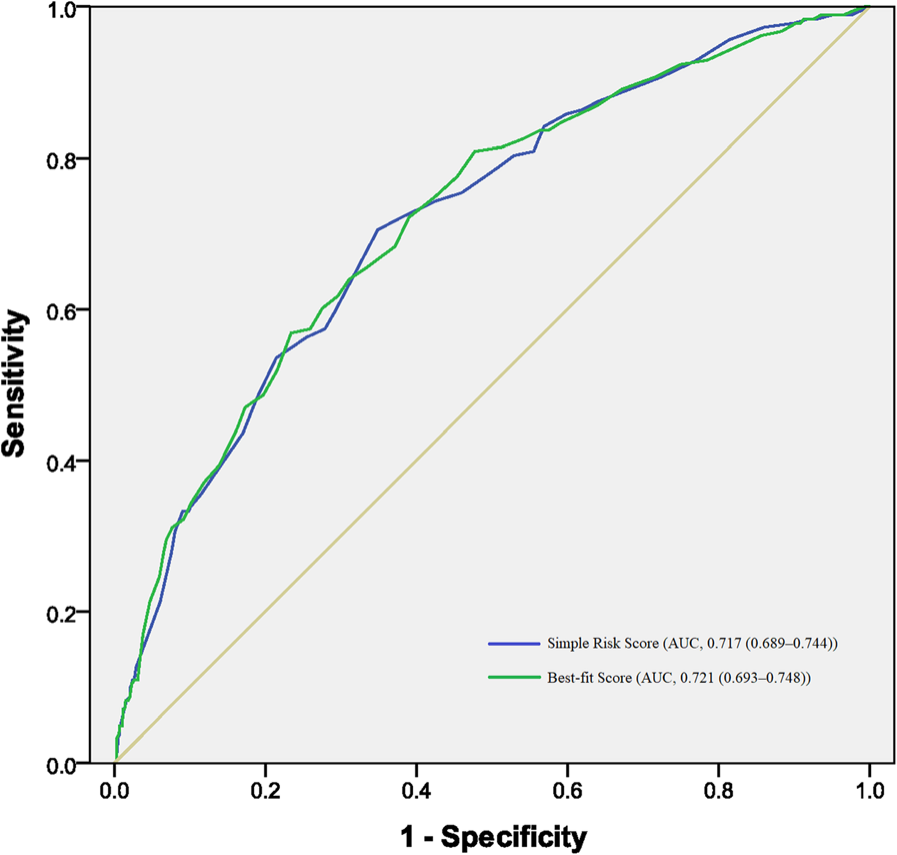
Receiver operating characteristic curves for the two new risk scores applied to the validation population

As shown in Tables 3 and 4, risk algorithms for the simple clinical model and the best-fit model were converted to risk scores based on the logistic regression coefficients and reference values for each significant risk factor. The Simple Risk Score included WC (4 points), SBP (23 points), sex (11 points), education (− 8 points), and diabetes (16 points) (Table 3). The Best-fit Risk Score included UACR (4 points), SBP (29 points), CRP (2 points), triglyceride (5 points), sex (13 points), education (− 10 points), and diabetes (18 points) (Table 4). As shown in Table 5 and Fig. 2, the values indicating the performance of the Simple Risk Score (derived from the simple clinical model) were: χ^2^ = 4.89, *P* = 0.769, AUC = 0.717 (95% CI, 0.689–0.744), *P* < 0.001, and for the Best-fit Risk Score (derived from the best-fit Model) were: χ^2^ = 2.52, *P* = 0.961, AUC = 0.721 (95% CI, 0.693–0.748), *P* < 0.001.

**Table 3:** Algorithm for estimating the risk of CKD using total point values in the simple model

Risk factor	Reference value (W_**ij**_)	β_**i**_	β_**i**_ (W_**ij**_-W_**iREF**_)	Point_**ij**_ = β_**i**_ (W_**ij**_-W_**iREF**_)/B^a^
**Waist circumference, men/women (cm)**		0.013		
< 80/75	77.0/72.0(W_1REF_)		0	0
80–84.9/75–79.9	82.5/77.5		0.072	1
85–89.9/80–84.9	87.5/82.5		0.137	2
90–94.9/85–89.9	92.5/87.5		0.202	3
≥ 95/90	99.0/95.0		0.286	4
**SBP (mmHg)**		0.025		
< 120	110(W_2REF_)		0	0
120–139	130		0.500	8
140–159	150		1.000	15
> 160	170		1.500	23
**Sex**		0.686		
Men	1(W_3REF_)		0	0
Women	2		0.686	11
**Education**		−0.532		
Illiterate	1(W_4REF_)		0	0
Primary School and above	2		−0.532	−8
**Diabetes**		1.042		
NO	0(W_5REF_)		0	0
YES	1		1.042	16

^a^B = 5*0.013 = 0.065; CKD: Chronic kidney disease; SBP: systolic blood pressure

**Table 4:** Algorithm for estimating the risk of CKD using total point values in the best-fit model

Risk factor	Reference value (W_**ij**_)	β_**i**_	β_**i**_ (W_**ij**_-W_**iREF**_)	Point_**ij**_ = β_**i**_ (W_**ij**_-W_**iREF**_)/B^a^
**Urinary Albumin-to-creatinine ratio**		0.017		
< 5.0	2.68(W_1REF_)		0	**0**
5.0–10.0	7.16		0.076	1
> 10.0	15.71		0.222	4
**SBP (mmHg)**		0.025		
< 120	110(W_2REF_)		0	0
120–139	130		0.500	10
140–159	150		1.000	20
> 160	170		1.500	29
**CRP**		0.027		
< 1.0	0.40(W_3REF_)		0	0
1–3	1.73		0.036	1
> 3.0	4.66		0.115	2
**Triglycerides**		0.175		
< 1.0	0.77(W_4REF_)		0	0
1.0–1.7	1.30		0.093	2
> 1.7	2.26		0.261	5
**Sex**		0.652		
Men	1(W_5REF_)		0	0
Women	2		0.6520	13
**Education**		−0.516		
Illiterate	1(W_6REF_)		0	0
Primary School or more	2		−0.5160	−10
**Diabetes**		0.923		
NO	0(W_7REF_)		0	0
YES	1		0.9230	18

^a^B = 3*0.017 = 0.051; *CKD* Chronic kidney disease, *SBP* Systolic blood pressure, *CRP* C-reactive protein

**Table 5:** Performance of our risk scores in the prediction of incident CKD in the validation population

Scores	Risk factors in the score	AUC	Optimal cutoff value	Sensitivity	Specificity	+LR	-LR	+PV	-PV	Youden index	Hosmer and Lemeshow Test
Simple risk score	Sex, education, WC, diabetes, SBP.	0.717 (0.689–0.744)	> 14	70.49 (63.30–77.00)	65.14 (61.90–68.30)	2.02 (1.80–2.30)	0.45 (0.40–0.60)	29.8 (25.5–34.4)	91.3 (88.8–93.4)	0.3563	0.769
Best-fit risk score	Sex, education, diabetes, SBP, triglycerides, CRP, UACR.	0.721 (0.693–0.748)	> 24	56.83 (49.30–64.10)	76.61 (73.70–79.40)	2.43 (2.00–2.90)	0.56 (0.50–0.70)	33.8 (28.5–39.3)	89.4 (87.0–91.5)	0.3344	0.961

*CKD* Chronic kidney disease, *LR* Likelihood ratio, *PV* Predictive value, *WC* Waist circumference, *SBP* Systolic blood pressure, *CRP* C-reactive protein, *UACR* Urinary albumin-to-creatinine ratio

In the validation population, the difference in discrimination (AUC) between the Simple and the Best-fit Risk Score was not significant (*P* = 0.455). Compared with the best fitted risk score, when the optimal cut-off value was 14, the simple risk score had higher Youden index (0.3563), sensitivity (70.49%) and negative predictive value (91.3%). In addition, 66.6% of the participants had a risk ≤20.0, 28.9% had a risk > 20.0% but ≤40.0, and 4.5% had a risk > 40% using the Simple Risk Score system.

## Discussion

### Main findings

Using clinical demographic characteristics and laboratory information, we developed two risk scoring systems to predict the 5-year risk of incident CKD in a rural Chinese population of individuals aged 30 years and older. The Simple Risk Score was as useful as the Best-fit Risk Score for screening individuals at high-risk of developing CKD in a rural Chinese population. The Simple Risk Score is based on five clinical variables (sex, WC, SBP, diabetes, and education) and does not require blood or urine tests. In addition, the clinical variables in the scoring system can be easily obtained from families and health clinics, and it is also simple to use in rural China.

### Comparison with other risk scores

To our knowledge, several useful risk scores for predicting incident CKD have been developed in Western populations in developed areas, such as the United States and Europe [11, 13, 14]. In those studies, the prediction models for incident CKD showed that age, sex, diabetes, hypertension, CVD, eGFR, and albuminuria were associated with a risk of incident CKD with AUC values ranging from 0.70 to 0.88.

Asians have the highest prevalence of CKD worldwide [3] and the risk factors for incident CKD are different in Asian populations in developed versus low- and middle-income areas. In developed areas, such as in Japan [24], researchers found that older age, proteinuria, hematuria, higher SBP, taking antihypertensive and/or anti-diabetic medications, and current smoking were associated with and increased risk of CKD and higher eGFR and daily alcohol intake were associated with a lower risk. The C-statistics for the risk estimation equations for CKD at 10 years were > 0.8. In Taiwan [25], Chien and colleagues established a clinical prediction risk model based on age, BMI, DBP, and history of type 2 diabetes and stroke in a cohort study that had poor discriminatory power (c-statistic 0.67) and short-term follow-up (median 2.2 years). In a recent study conducted in low- to middle-income areas in Thailand [26], age, sex, SBP, diabetes, and WC were significant predictors for their clinical score, while age, sex, SBP, diabetes, and eGFR were predictors in the combined clinical and laboratory model. Both risk scores had a high degree of accuracy and discriminatory power in the Thai population (AUC 0.72–0.79). However, those two simple clinical risk scores that were derived from populations in Taiwan and Thailand performed poorly when tested in our cohort (AUC 0.615 and 0.621, respectively).

In our study, we developed two risk scores based on a general population living in a low- and middle-income rural area in Northern China. Compared with most previous studies, age was not highly associated with the risk of incident CKD in the present study. In our scoring system, SBP, diabetes, and sex were more important contributors to the overall score. Education was also an important clinical predictor of CKD, although it was not included in other scoring models developed in Western and other Asian populations. There are several explanations for the differences between our study and previous studies. First, we defined CKD as either reduced renal function or by the presence of albuminuria. In our study population, 95.8% were identified by the presence of albuminuria and 6.4% were identified by reduced renal function (eGFR < 60 mL/min/1.73 m^2^). Second, China is the world’s largest developing country with a rapidly increasing prevalence of diabetes, hypertension, and obesity [2, 15, 27]. The prevalence rates of hypertension, diabetes, and obesity in our rural population were 45.6, 4.9, and 13.9%, respectively; however, the percentages of use of antihypertensive and anti-diabetic medications were only 40.3 and 27.3%, respectively. Third, 12.2% of our participants were illiterate and 52.2% were educated to the primary school level; thus, in our study, education levels were negatively associated with a risk of CKD. Finally, compared with men, women had higher BMIs and SBPs but lower education levels and were less physically active.

### Strengths and limitations

To the best of our knowledge, this is the first study to develop simple risk scores for predicting incident CKD in a population living in a low- and middle-income rural area of China. Further, this study was a village-based cohort study, with a detailed assessment of risk factors including measures of baseline renal function and albuminuria. However, there were also several limitations to this study. First, a total of 6830 participants participated in the village-based cohort study from 2006 to 2013; however, only 3266 participants were included in our final analysis, as 3564 participants were excluded for various reasons. Therefore, there may be selection bias. Additionally, compared with previous studies [24–26], this study is relatively small. Second, external validation has not been carried out because there are no data available from other similar studies in China. Third, a family history of kidney disease may be associated with CKD, but questions to identify this information were not addressed in our questionnaires. Fourth, the discriminatory capacity of our Simple Risk Score was moderate (the AUC was 0.717) and somewhat lower than that of other risk scores developed in other populations. Finally, participants with acute renal injury was not ruled out due to the lack of creatinine data for the most recent week at the time of the survey. Moreover, the present scoring system is based on the data of HES from 2006 to 2013, so we must carefully apply these results to the current management of CKD high-risk population.

## Conclusions

In this cohort study, we developed our Simple Risk Score for predicting incident CKD based upon age, sex, SBP, diabetes, and WC. In China, screening for CKD should be a priority in low- and middle-income areas, because early intervention is likely to be effective in reducing the high morbidity and mortality rates resulting from CKD. This simple CKD scoring system can be integrated into the rural primary health care system and help to screen and identify high-risk individuals of incident CKD. This will be particularly beneficial for women with hypertension, overweight, and those with low education levels in rural areas. It is anticipated that this scoring system will improve CKD prevention and provide necessary information for the implementation of intervention strategies among rural populations in China.

## Data Availability

The datasets used and analysed during the current study are available from the corresponding author on reasonable request.

## Abbreviations

CKD: Chronic kidney disease
eGFR: Estimated glomerular filtration rate
UACR: Urinary albumin-to-creatinine ratio
CI: Confidence interval
CVD: Cardiovascular disease
HES: Handan eye study
SBP: Systolic blood pressure
DBP: Diastolic blood pressure
BMI: Body mass index
WC: Waist circumference
FPG: Fasting plasma glucose
AUC: Area under the receiver operating characteristic curve
CRP: C-reactive protein
ROC: Receiver operating characteristic curve
